# *BUB1*, *BUB1B*, *CCNA2*, and *CDCA8*, along with miR-524-5p, as clinically relevant biomarkers for the diagnosis and treatment of endometrial carcinoma

**DOI:** 10.1186/s12885-023-11515-9

**Published:** 2023-10-18

**Authors:** Qirong Hao, Hongqin Wu, Erniao Liu, Lina Wang

**Affiliations:** Department of Obstetrics and Gynecology, the Second Hospital of Shanxi Medical University, Taiyuan, 030001 China

**Keywords:** miR-524-5p, Endometrial carcinoma (EC), Ishikawa cells, biomarker

## Abstract

**Background:**

Endometrial carcinoma (EC) is a malignant tumor of the female reproductive tract that has been associated with increased morbidity and mortality. This study aimed to identify biomarkers and potential therapeutic targets for EC.

**Methods:**

A publicly available transcriptome data set comprising 587 EC cases was subjected to a comprehensive bioinformatics analysis to identify candidate genes responsible for EC occurrence and development. Next, we used clinical samples and cell experiments for validation.

**Results:**

A total of 1,617 differentially expressed genes (DEGs) were identified. Analysis of patient survival outcomes revealed that *BUB1*, *BUB1B*, *CCNA2*, and *CDCA8* were correlated with prognosis in patients with EC. Moreover, assessment of clinical samples confirmed that BUB1, BUB1B, CCNA2 and CDCA8 were strongly expressed in EC tissues. Additionally, bioinformatics and luciferase reporter assays confirmed that miR-524-5p can target and regulate these four genes. Overexpression of miR-524-5p significantly inhibited EC Ishikawa cells viability, migration and invasion. Inhibition of miR-524-5p showed the opposite results.

**Conclusions:**

Expression of miR-524-5p reduced the migration and invasion of Ishikawa EC cells, and decreased *BUB1*, *BUB1B*, *CCNA2*, and *CDCA8* expression. miR-524-5p, as well as *BUB1*, *BUB1B*, *CCNA2*, and *CDCA8*, may be clinically relevant biomarkers for the diagnosis and treatment of EC.

**Supplementary Information:**

The online version contains supplementary material available at 10.1186/s12885-023-11515-9.

## Highlight Box

### Key findings

The expression of microRNA (miR)-524-5p reduced the migration and invasion of Ishikawa endometrial carcinoma (EC) cells, while decreasing *BUB1*, *BUB1B*, *CCNA2*, and *CDCA8* expression.

### What is known and what is new?

*miR-524-5p*, encoded on chromosome 19q13.42, was found to promote osteosarcoma by regulating the deletion of the phosphatase and tensin homolog (*PTEN*) gene located on chromosome 10. *miR-524-5p* reduced the migration and invasion of EC cells and the expression of *BUB1*, *BUB1B*, *CCNA2*, and *CDCA8*.

### What is the implication, and what should change now?

*miR-524-5p* can regulate *BUB1*, *BUB1B*, *CCNA2*, and *CDCA8*, thereby contributing to the development of EC. Thus, miR-524-5p, as well as *BUB1*, *BUB1B*, *CCNA2*, and *CDCA8*, may be clinically relevant biomarkers for the diagnosis and treatment of EC.

## Introduction

Endometrial carcinoma (EC) is a malignant tumor of the female reproductive tract that has been associated with increased morbidity and mortality. In 2018, an estimated 61,380 new cases were reported in the United States alone, resulting in 11,350 deaths [1, 2]. A steady rise in EC incidence has been observed and is expected to continue [3]. Data from the Surveillance, Epidemiology, and End Results program showed that women with stage I endometrioid cancer have a 5-year survival of > 98% [4, 5]; however, patients with advanced, relapsed, or metastatic EC usually develop other complications and are unable to undergo radical EC surgery [6], resulting in a considerably reduced survival rate. Therefore, new diagnostic and prognostic biomarkers are urgently needed to expedite the development of specific and efficient treatments for EC.

ECs can be classified into EC and other cancers, including mucosal adenocarcinoma, adenocarcinoma in the genitalia, simple melanoma, melanoma, and mixed cancer. A small number of cancers show interstitial differentiation and are called carcinosarcomas. Among newly diagnosed cases, EC accounts for more than 85% of cases, serous carcinoma for 3–10%, clear cell carcinoma for 2–3%, and carcinosarcoma for less than 2%, with other tissue types accounting for an even lower proportion [7]. The European Society of Oncology identifies the risk of EC based on the patient’s age, cancer histological type, degree of tumor, lymphovascular space invasion (LVSI), and cervical invasion [8]. The development of sequencing technology has led to the detection of several differentially expressed genes (DEGs) between EC and normal endometrial tissues. Based on this, endometrial cancer is now classified according to the presence of DNA polymerase (POLE) hypermutation, unstable microsatellites, and ultra-low and -high copy number types, which have replaced the traditional binary model of endometrioid and non-endometrioid classification. These DEGs can also be taken as biological engines for EC risk assessment and prognosis [9–11]. Therefore, investigations on candidate genes may help to further understand EC pathogenesis, thereby improving the diagnostic accuracy and treatment effectiveness.

Several cancer biomarkers have been discovered by bioinformatics, with consequent increases in the number of novel strategies and drugs used for cancer diagnosis, treatment, and prognosis [12]. These results have yielded valuable biological information and provided important research tools for finding specific and sensitive diagnostic and prognostic molecular markers [13, 14]. Moreover, recent cancer studies have applied comprehensive bioinformatics methods to overcome the limitations and data variability of the available reports [15, 16]. The Cancer Genome Atlas (TCGA) database is a major source of valuable experimental data on the DNA, RNA, protein, and epigenetic level of a large number of human tumor tissues [17].

MicroRNAs (miRNAs) are short (18–25 nucleotides), noncoding RNA sequences. The human genome encodes thousands of miRNAs that control more than 30% of the genome, thus contributing to almost all the basic cellular functions, including differentiation, growth, migration, and apoptosis [18, 19]. The occurrence and development of ECs are closely related to abnormal miRNA expression. Recently, miR-524-5p, encoded on chromosome 19q13.42, was reported to promote osteosarcoma by regulating the deletion of the phosphatase and tensin homolog (*PTEN*) gene located on chromosome 10 [20]. *miR-524-5p*, as a cancer-suppressor gene, was found to inhibit thyroid cancer proliferation by regulating *SPAG9* [21, 22].

In this study, several bioinformatics methods were used, such as including the “DESeq” and “edgeR” analysis tools in R, Gene Ontology (GO) analysis, and protein-protein interaction (PPI) network analysis, to screen DEGs based on TCGA data of 587 cases of EC. Then we performed histological verification and cellular level verification on the pathological sections of EC patients. This study provides a foundation for identifying novel EC biomarkers and therapeutic targets.

## Methods

### EC transcriptome chipset

EC messenger RNA (mRNA) chip expression profile data were obtained from the TCGA database (https://portal.gdc.cancer.gov/; accessed at 2022.05.01). The search terms used were “Uteri”, “TCGA-UCEC”, “Transcriptome Profiling”, “Gene Expression Quantification”, and HTSeq-Counts”. Among the results obtained, 35 non-cancer cases were used as the control group, whereas data from 552 EC cases were used as the experimental group. A noncoding RNA profile data set (GSE25405), including 7 control groups and 20 endometrial cancer tissues, was obtained from the Gene Expression Omnibus (GEO). Next, differential gene analyses were performed based on the disease transcriptome data.

### Differential gene analysis

The TCGA expression data set was corrected and analyzed using the “edgeR” [23] and “DESeq” [24] packages of the R software (https://cran.r-project.org/). The “pheatmap” package was used to draw a heat map of the identified DEGs [25], in which the intersection, as determined by the cutoff value of log fold change (FC) ≥ 1 or ≤ − 1 and *P* value < 0.05 represented the candidate DEGs.

### GO and Kyoto Gene and Genome Encyclopedia (KEGG) pathway analysis of DEGs

The Database for Annotation, Visualization, and Integrated Discovery (DAVID) gene annotation online tool was used to facilitate the identification of the biological function of the DEGs in EC. GO analysis using a false discovery rate < 0.05 was performed to assess the enrichment function of DEGs, and the R “ggplot2” package was used for mapping [26]. KEGG pathway analysis (www.kegg.jp/kegg/kegg1.html) was performed via the “clusterProfiler” package in R [27] with a hypergeometric distribution for functional classification and enrichment of gene clusters under the condition of a *P* value < 0.01 [28].

### Construction of the PPI network, hub gene screening, and survival analysis

The Search Tool for the Retrieval of Interacting Genes/Proteins (STRING) database was used to construct the PPI network of the DEGs and analyze the functional interactions between proteins to help clarify the mechanism of disease onset and development [29, 30]. We used the Maximum Neighborhood Component analysis tool of Cytoscape in cytoHubba [31] to find the hub genes of the densely connected regions of topological clustering. The “survival” package in R [32] was then used for survival analysis related to the identified hub genes based on the transcriptome data and survival data of the TCGA EC data set.

### Patient enrolment

Between January 2016 and May 2022, 79 patients were recruited, including 29 cases of uterine fibroids and 50 cases of EC. Diagnosis and staging were performed according to the International Federation of Gynecology and Obstetrics (FIGO) 2014 surgical pathological staging guidelines [33]. In all cases, the uterus was surgically extracted, and tissue samples were fixed and embedded in paraffin. The exclusion criteria were as follows: pregnant women and those with other malignant tumors or other complications, such as diabetes and hypertension. The study was conducted in accordance with the Declaration of Helsinki (as revised in 2013). The study was approved by the Ethics Committee of the Second Hospital of Shanxi Medical University board of No. 2021YX135 and informed consent was taken from all the patients.

### Immunohistochemistry

Paraffin-embedded, 5-µm-thick sections were dewaxed in xylene, eluted with a gradient of ethanol to water, blocked with 3% H_2_O_2_ for 10 min, and added to antigen repair solution [0.01 M under high pressure with citrate buffer (pH 6.0)]. After the sections were cooled to room temperature for 2.5 min, a blocking solution was added dropwise. These sections were incubated with anti-cyclin A2 (CCNA2) (1:1,000; Abcam, Cambridge, UK), anti-BUB1 mitotic checkpoint serine/threonine kinase (BUB1) (1:50; Abcam), anti-BUB1 mitotic checkpoint serine/threonine kinase B (BUB1B) (1:200; Abcam), and anti-cell division cycle associated 8 (CDCA8) (1:100; Abcam) antibodies overnight at 4 ℃, which was followed by a horseradish peroxidase-conjugated secondary antibody (1: 5,000, Abcam) added dropwise. 3,3’-diaminobenzidine was used for color development, with a positive result showing brownish yellow. A vertical microscope (BX53; Olympus, Tokyo, Japan) was used to collect images, and their analysis was performed using the Image-Pro Plus 6.0 software (Media Cybernetics, Rockville, MD, USA).

### Cell culture and miRNA transfection

Human endometrial epithelial cells (hEEC) and human endometrial cancer Ishikawa (ISK) cells (American Type Culture Collection, Manassas, VA, USA) were cultured in Dulbecco’s Modified Eagle Medium/Nutrient Mixture F-12 (DMEM/F-12; Boster Bio, Pleasanton, CA, USA) supplemented with 10% fetal bovine serum (New England Biolabs, Ipswich, MA, USA), 100 mg/mL of streptomycin, and 100 U/mL of penicillin under the conditions of relative humidity, 5% CO_2_, and 37 ℃. The cells were collected when 80% confluency was reached and used for subsequent analysis and experiments. Cells (4 × 10^5^/well) were seeded in 6-well plates in complete DMEM/F-12 and incubated for 24 h at 37 ℃. Next, 10 µL of miRNA mimics or inhibitor was transfected into ISK cells using Lipofectamine 2000 reagent (Invitrogen, Thermo Fisher Scientific, Waltham, MA, USA) according to the manufacturer’s protocol. The cells were then incubated at 37 ℃ for 6 h. Afterward, the culture medium was removed and replaced by complete culture medium, and the cells were incubated at 37 ℃ for an additional 24 h. A micrograph was taken with an Olympus BX53 microscope.

### MTT assay

ISK cells were seeded in 96-well plates (5 × 10^3^ cells/well) and incubated overnight at 37 ℃. A blank control, miRNA inhibitor, and miRNA mimic, as well as the respective negative controls (NCs) were transfected into ISK cells (as described in Sect. 2.7) and cultured for 48 h. MTT reagent (10 µL; Solarbio, Beijing, China) was added to each well and cultured at 37 ℃ for 4 h. Then, the culture medium was removed and 110 µL of formazan was added and incubated for 10 min. The absorbance of each well at 490 nm was determined using a microplate reader.

### Dual-luciferase reporter gene assay

The ENCORI database (http://starbase.sysu.edu.cn/index.php) was used to predict the binding sites of miR-524-5p and BUB1, miR-524-5p and BUB1B, miR-524-5p and CCNA2, miR-524-5p and CDCA8 (Supplementary Fig. 1). Meanwhile, dual luciferase assay was used to verify the targeting relationship between miR-524-5p and BUB1, miR-524-5p and BUB1B, miR-524-5p and CCNA2, and miR-524-5p and CDCA8 in Ishikawa cells [34].

### Wound healing assay

miRNA-transfected, miRNA-NC-transfected, and non-transfected ISK cells (4 × 10^5^ cells/well) were seeded in complete culture medium in 6-well plates previously marked with a horizontal line drawn evenly (about 0.5 to 1 cm) in each well. When the cells reached 90% confluency, a scratch was made to the cell layer with a pipette tip. The cells were washed thrice with phosphate-buffered saline (PBS) and photographed for 24 h at 0 ℃ using an inverted microscope (Olympus). Cell migration potential was determined based on width changes of the scratch of the control and experimental groups and as measured using ImageJ and software (US National Institutes of Health, Bethesda, MD, USA) as per the following equation: migration distance (%) = [(At_0_ – At_c_)/At0] ×100, in which At_0_ and At_c_ represent the average scratch width at 0 and 24 h, respectively. All experiments were repeated 3 times.

### ISK cell migration assay

miRNA-transfected ISK cells (2 × 10^5^ cells/mL) were seeded in Transwell chambers placed on 24-well plates (Corning, Corning, NY, USA). After 1 h, approximately 200 cells from the cell suspension from each group were placed on the upper chamber of the Transwell and incubated in a 5% CO_2_ incubator at 37 ℃ for 24 h. Next, the Transwell chamber was carefully removed and washed with PBS, and the cells were fixed with 70% ice-cold ethanol solution for 1 h and stained with 0.5% crystal violet for 20 min at room temperature. The cells were washed again with PBS, and the top of the chamber was washed with a clean cotton ball. The cells in the bottom of the chamber were examined, photographed, and counted under the microscope. All experiments were performed 3 times.

### ISK cell invasion assay

Transwell chambers were coated with 100 µL of Matrigel (1 mg/mL; Corning) and inserted into 24-well plates with complete medium in the bottom of the well. After the Matrigel layer was formed (at 37 ℃ for about 4–5 h), 200 µL of miRNA-transfected ISK cells (2.5 × 10^5^/mL) were placed in the upper chamber of the Transwell and incubated at 37 ℃ for 24 h. Next, the well was carefully removed and washed with PBS, and the cells were fixed with 70% cold ethanol solution for 1 h and stained with 0.5% solution of crystalline purple dye at room temperature for 20 min. The cells were rinsed with PBS, and the top of the chamber was cleaned with a clean cotton swab to remove the noninvasive cells. The Transwell Matrigel membrane was then observed under an inverted microscope and photographed to determine the number of invasive cells. All the experiments were repeated thrice.

### RNA extraction, reverse transcription, and quantitative real-time polymerase chain reaction analysis

Total RNA was isolated using TRIzol (Vazyme Biotech, Nanjing, China), immediately resuspended in MilliQ water, and stored at − 80 ℃ until use. The quantity and purity of the RNA samples were determined using a NanoDrop Spectrophotometer (Thermo Fisher Scientific, Waltham, MA, USA). Complement DNA (cDNA) was synthesized from 1 µg of RNA sample using a cDNA synthetic kit (Tiangen Biotech, Beijing, China). Next, cDNA (20 ng) was used to amplify the reference genome (*U6*) or target gene (*miR-524-5p*) using specific primer sets, with their amplification efficiency of close to 100% (*U6*, forward primer: 5’-CGCTTCGGCAGCACATATAC-3’; reverse primer: 5’-AAATATGGAACGCTTCACGA-3’; *miR-524-5p*, loop primer: 5’-GTCGTATCCAGTGCAGGGTCCGAGGTATTCGCACTGGATACGACGAGAAAGT-3’; forward primer: 5’-TGCGCCTACAAAGGGAAGCACT-3’). The 20-µL reaction mixture consisted of 4 µL of cDNA, 0.4 µL (10 µM) of forward and reverse primers, 4.8 µL of RNase/DNase-free water, 10 µL of SYBR green buffer (Vazyme Biotech), and 0.4 µL of 50 × ROX (Vazyme Biotech). The ABI QuantStudio 6 (Thermo Fisher Scientific) was used for quantitative real-time polymerase chain reaction (qRT-PCR) analysis of mRNA levels. The qRT-PCR conditions were as follows: Taq was thermally activated and denatured at 95 ℃ for 2 min, which was followed by 40 cycles of 95 ℃ for 10 min, 95 ℃ for 30 s, and 60 ℃ for 30 s. Gene expression was calculated using the 2^−ΔΔCt^ method, and all experiments were repeated 3 times.

### Western blot

Total protein was extracted from cells with RIPA lysate and the protein concentration was determined by BCA assay. Protein samples were separated by 10% SDS-polyacrylamide gel electrophoresis and transferred to polyvinylidene fluoride (PVDF) membranes (Millipore, Bedford, MA, USA). The film was enclosed in 5% skim milk powder at room temperature for 2 h, and then incubated with primary antibody anti-Bub1 antibody (ab195268), Bub1b antibody (#4116, Cell Signaling Technology), anti-Cyclin D2 antibody (ab230883), anti-CDCA8 antibody (ab67126) at 4℃ overnight. After TBST washing 3 times, they were incubated with anti-rabbit or anti-mouse IgG coupled with horseradish peroxidase. The film was immersed in the enhanced chemiluminescence solution for 1 min, and the protein bands were exposed and developed using the Bio-Rad image lab software. The relative expression levels were normalized with GAPDH. The images were analyzed using ImageJ2x software (National Institutes of Health (NIH), Maryland, USA).

### Statistical analysis

The Kaplan-Meier method was used to analyze the survival of patients according to gene expression patterns. “edgeR” in R used the conditional maximum likelihood condition (based on the total count of the gene) to calculate the dispersion of the gene, and the empirical Bayesian method was used to reduce the dispersion of the gene to the common dispersion. Finally, a precise test similar to the Fisher exact test was used to evaluate the differential expression of each gene. “DESeq” in R used the size factor to normalize the data (the median ratio of the observed count) to make the samples comparable when these samples were sequenced at different depths [35]. A random 2-sample *t*-test was used to analyze gene expression between tumor and normal samples as well as between tumor samples and benign uterine diseases. One-way analysis of variance was performed according to the Dunnett test to compare the differences between groups. The minimum PPI interaction score was 0.4000. SPSS 20.0 (IBM Corp., Armonk, NY, USA) was used to perform the statistical analyses. *P* < 0.05 was considered to indicate statistical significance as appropriate.

## Results

### Differential gene screening

**Fig. 1:**
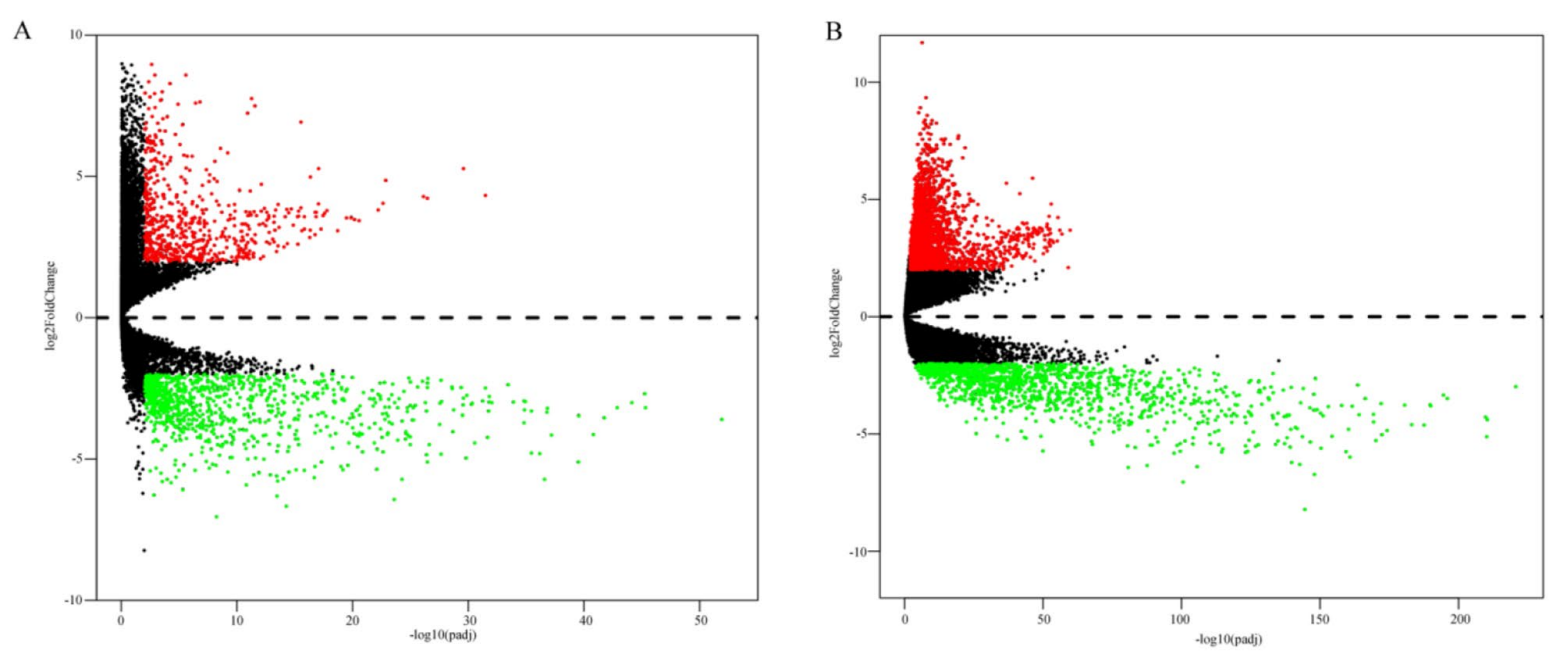
Identification of DEGs associated with endometrial cancer. (**A**) Volcano map for DEGs identified using DESeq. (**B**) Volcano map for DEGs identified using edgeR. A gradual change in color from green to red indicates the gene expression transition from downregulated to upregulated. Endometrial cancer cases: 552; non-cancer cases: 35. DEGs, differentially expressed genes

**Fig. 2:**
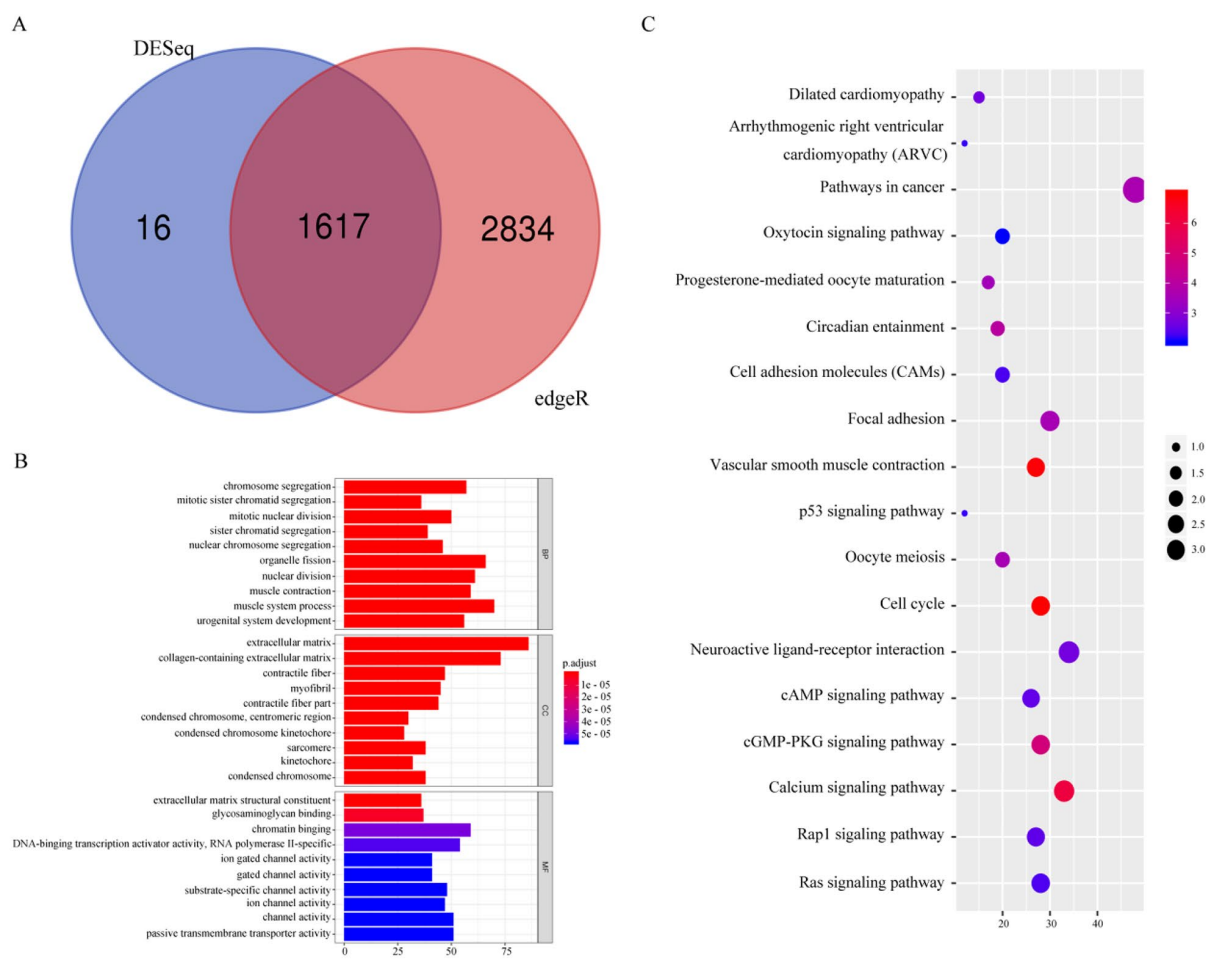
Differential GO analysis and KEGG pathway analysis. (**A**) Venn diagrams showing 1617 intersecting DEGs from DESeq and edgeR screening. (**B**) GO enrichment analysis of the intersecting DEGs. The y-axis labels represent the aggregated GO terms. (**C**) KEGG pathway analysis of the intersecting DEGs. The y-axis labels represent the clustered KEGG pathway. GeneRatio represents the ratio of the number of genes enriched in a KEGG pathway to the number of DEGs that were up- or downregulated. GO, gene ontology; KEGG, kyoto encyclopedia of genes and genomes; DEGs, differentially expressed genes

Differential gene analysis using the R packages “DESeq” and “edgeR” on TCGA gene expression data set identified 1,633 and 4,451 DEGs, respectively (Fig. 1A and B), and a Venn plot revealed 1,617 common DEGs (Fig. 2A).

### Biological function analysis of DEGs

GO functional annotation of the 1,617 DEGs using the DAVID online tool revealed that these genes were mainly related to chromosome segregation, mitotic nuclear division, nuclear division, sarcomere, channel activity, and calcium ion binding, among others (Fig. 2B). The “clusterProfiler” analysis further showed that the role of DEGs in EC was mainly focused on the cell cycle, calcium signaling pathway, cGMP-PKG signaling pathway, cancer pathway, cAMP signaling pathway, Rap1 signaling pathway, Ras signaling pathway, P53, and other signaling pathways (Fig. 2C).

### Hub gene screening and prognostic analysis

**Fig. 3:**
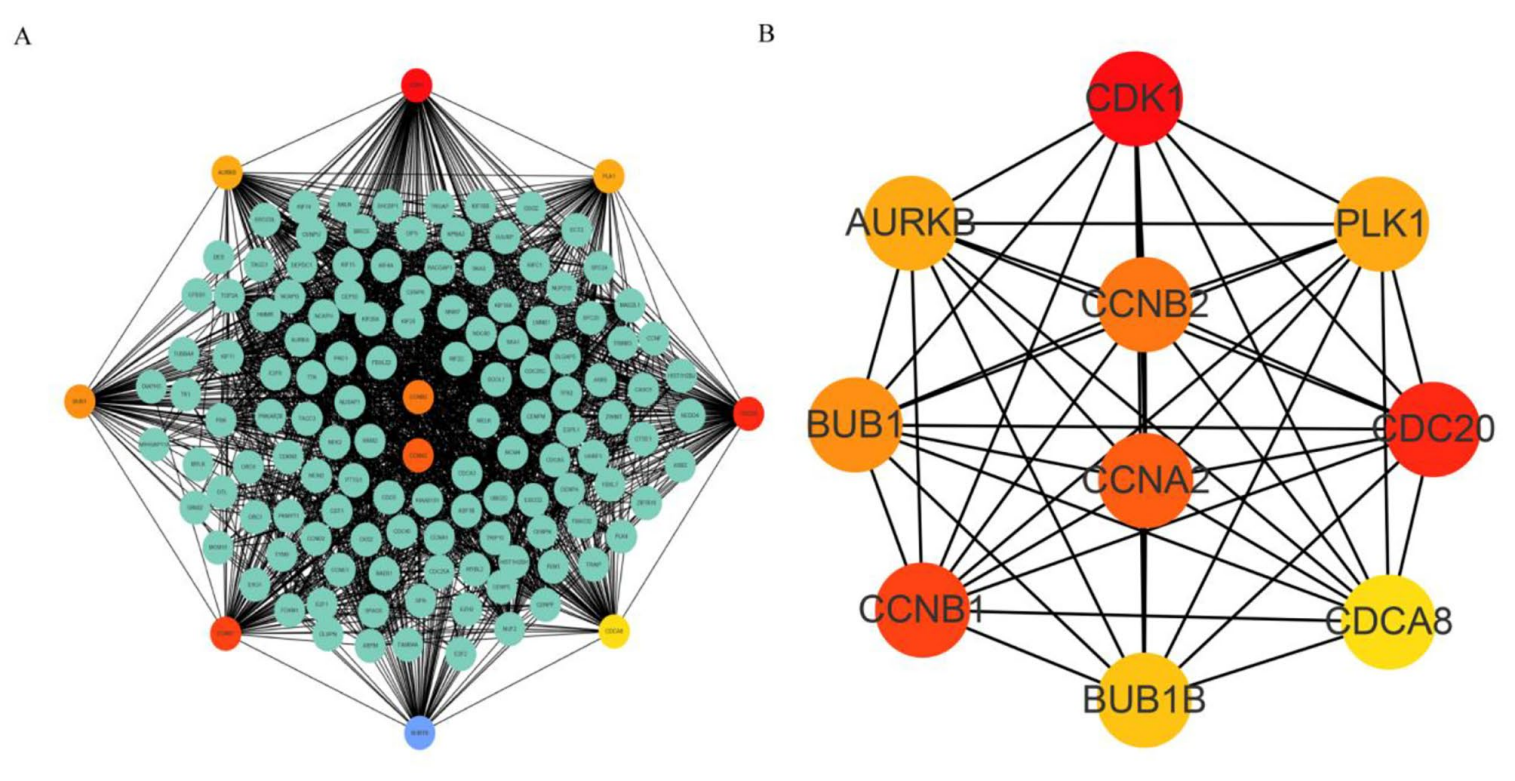
Interaction network of central genes and proteins. (**A**, **B**) Central gene network determined using Cytoscape’s cytoHubba. The coefficients for |r| are > 0.4 and *P* < 0.05

**Fig. 4:**
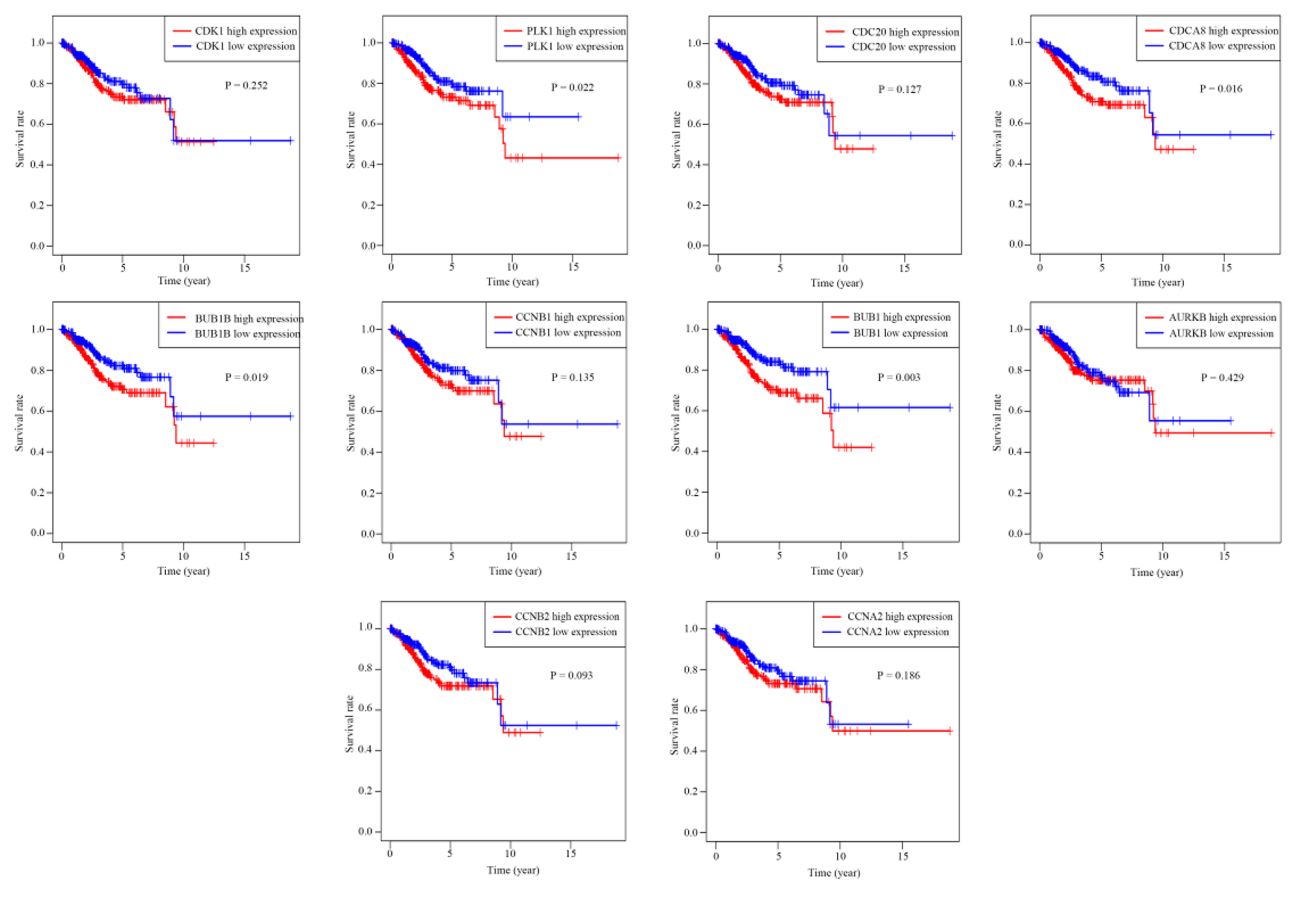
Survival analysis of 10 genes. The Kaplan-Meier curve shows the relationship between 10 genes and the survival of EC patients. *BUB1*, *BUB1B*, *PLK1*, and *CDCA8* were significantly related to the survival of EC patients. Patients with high *AURKB*, *CCNB1*, *CCNB2*, and *CCNA2* expression in conjunction with high CDC20 and CDK1 expression showed poor prognosis, but it was not statistically significant. *P* < 0.05 indicates a statistically significant difference. Endometrial cancer cases: 552

A PPI network of the EC-related DEGs was composed of 1,319 and 13,606 nodes and edges, respectively (Fig. 3A). The top 10 genes (*AURKB, CCNB1, CDCA8, CCNB2, BUB1, BUB1B, PLK1, CCNA2, CDC20*, and *CDK1*) were considered as hub genes (Fig. 3B). Kaplan-Meier analysis of these 10 genes revealed that *BUB1*, *BUB1B*, *PLK1*, and *CDCA8* were correlated with the prognosis of patients with EC and that those exhibiting high expression of these genes had a poor prognosis (*P* < 0.05). Additionally, patients with high expression of *AURKB*, *CCNB1*, *CCNB2*, and *CCNA2* along with high expression of *CDC20* and *CDK1* showed poor prognosis (Fig. 4), but it was not statistically significant (*P* > 0.05).

### Mir-524-5p targeted EC hub genes

**Fig. 5:**
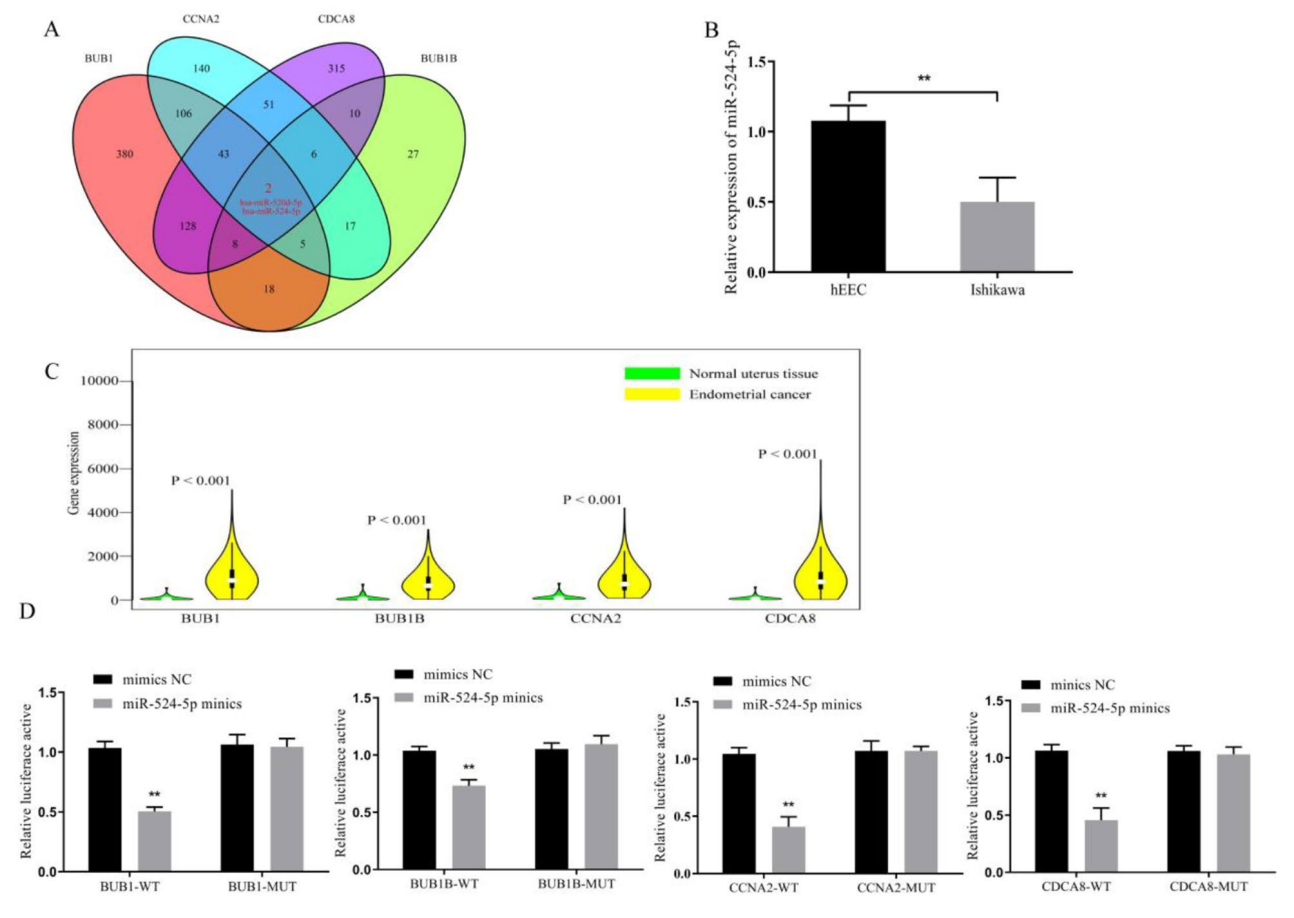
miR-524-5p targeted BUB1, BUB1B, CCNA2, and CDCA8. (**A**) TargetScan predicted *BUB1*, *BUB1B*, *CCNA2*, and *CDCA8* as the downstream target genes of miR-524-5p. (**B**) The miR-524-5p expression was detected by RT-qPCR in human normal endometrial cells (hEEC) and human endometrial cancer Ishikawa cells. (**C**) In TCGA database, *BUB1*, *BUB1B*, *CCNA2*, and *CDCA8* were all increased in endometrial cancer tissues, compared with normal tissues. (**D**) Dual luciferase assay was used to detect the targeting of miR-524-5p with BUB1, BUB1B, CCNA2, and CDCA8 in Ishikawa cells. **P < 0.01

The study employed TargetScan (https://www.targetscan.org/vert_72/) to screen for miRNAs regulating BUB1, BUB1B, CCNA2, and CDCA8. Subsequently, miRNAs (hsa-miR-520d-5p and hsa-miR-524-5p) that intersected with all four genes, BUB1, BUB1B, CCNA2, and CDCA8, were selected. Next, the GSE25405 dataset was used to identify differentially expressed miRNAs. Finally, hsa-miR-524-5p was the intersection of the common miRNAs of BUB1, BUB1B, CCNA2, and CDCA8 with the differentially expressed miRNAs from GSE25405 (Fig. 5A). RT-qPCR detection showed that the expression level of miR-524-5p in Ishikawa cells was significantly decreased compared with in the hEEC (*P* = 0.0081; Fig. 5B). Meanwhile, the expression of these genes was evaluated in 587 EC cases and their non-tumor counterparts with tissue microarray analysis via TCGA. The results showed that *BUB1*, *BUB1B*, *CCNA2*, and *CDCA8* were strongly expressed in EC tissues (*P <* 0.05; Fig. 5C). In addition, dual luciferase assay showed that there was a targeting relationship between miR-524-5p and BUB1, miR-524-5p and BUB1B, miR-524-5p and CCNA2, and miR-524-5p and CDCA8 in Ishikawa cells (Fig. 5D).

### Expression of BUB1, BUB1B, CCNA2, and CDCA8 in cancerous and noncancerous tissues

**Fig. 6:**
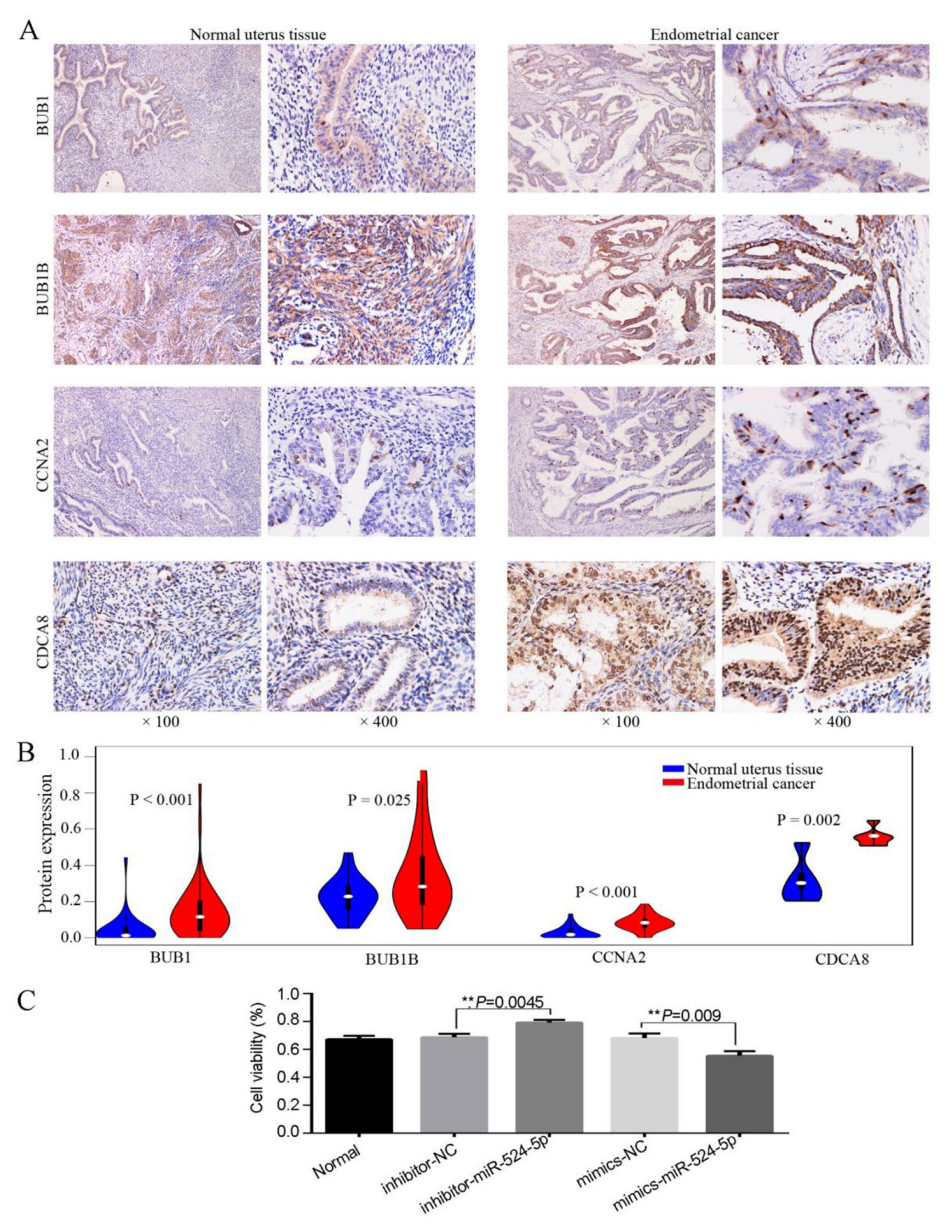
Analysis of central gene expression in 79 samples and TCGA database. (**A**, **B**) Compared with the normal group, endometrial cancer tissue showed high BUB1, BUB1B, CCNA2, and CDCA8 protein expression. *P* < 0.05 indicates a statistically significant difference. Endometrial cancer cases: 50; normal uterus tissue: 29. (**C**) Effects of miR-524-5p mimics and inhibitors in the viability of ISK cells. ISK, Ishikawa

A comprehensive bioinformatics analysis of endometrial cancer revealed 4 differential genes related to survival: *BUB1, BUB1B, CCNA2*, and *CDCA8*. Immunohistochemical experiments on tissues from 29 patients with uterine fibroids and 50 patients with EC were performed to confirm these findings. The results showed that the protein expressions of BUB1 (*P* < 0.001), BUB1B (*P* = 0.025), CCNA2 (*P* < 0.001), and CDCA8 (*P* = 0.002) were significantly up-regulated in the cancer tissues (Fig. 6A and B).

### Transfection of mir-524-5p into ISK cells reduced cell viability, wound healing, migration, and invasion potential

**Fig. 7:**
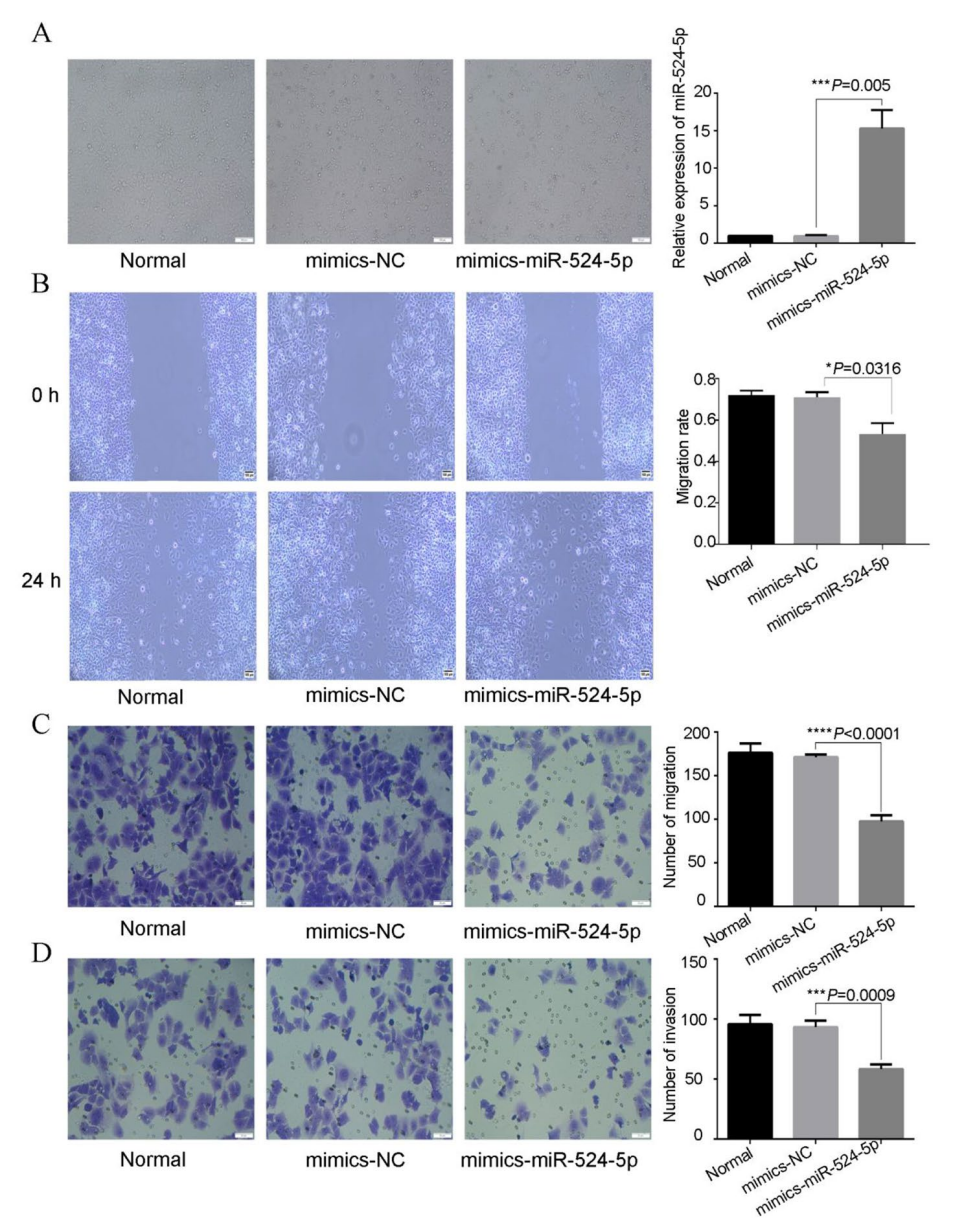
Effect of miR-524-5p on wound healing, migration, and invasion of ISK cells. (**A**) Transfection with miR-524-5p mimic resulted in the overexpression of miR-524-5p in ISK cells. miR-524-5p overexpression reduced the (**B**) wound healing capacity, (**C**) migration ability, and (**D**) invasion ability of ISK cells. *P <* 0.05 indicates a statistically significant difference. Data are expressed as the mean ± SD of three independent experiments. The number of cells: 4 × 10^5^/well. ISK, Ishikawa; SD, standard deviation

**Fig. 8:**
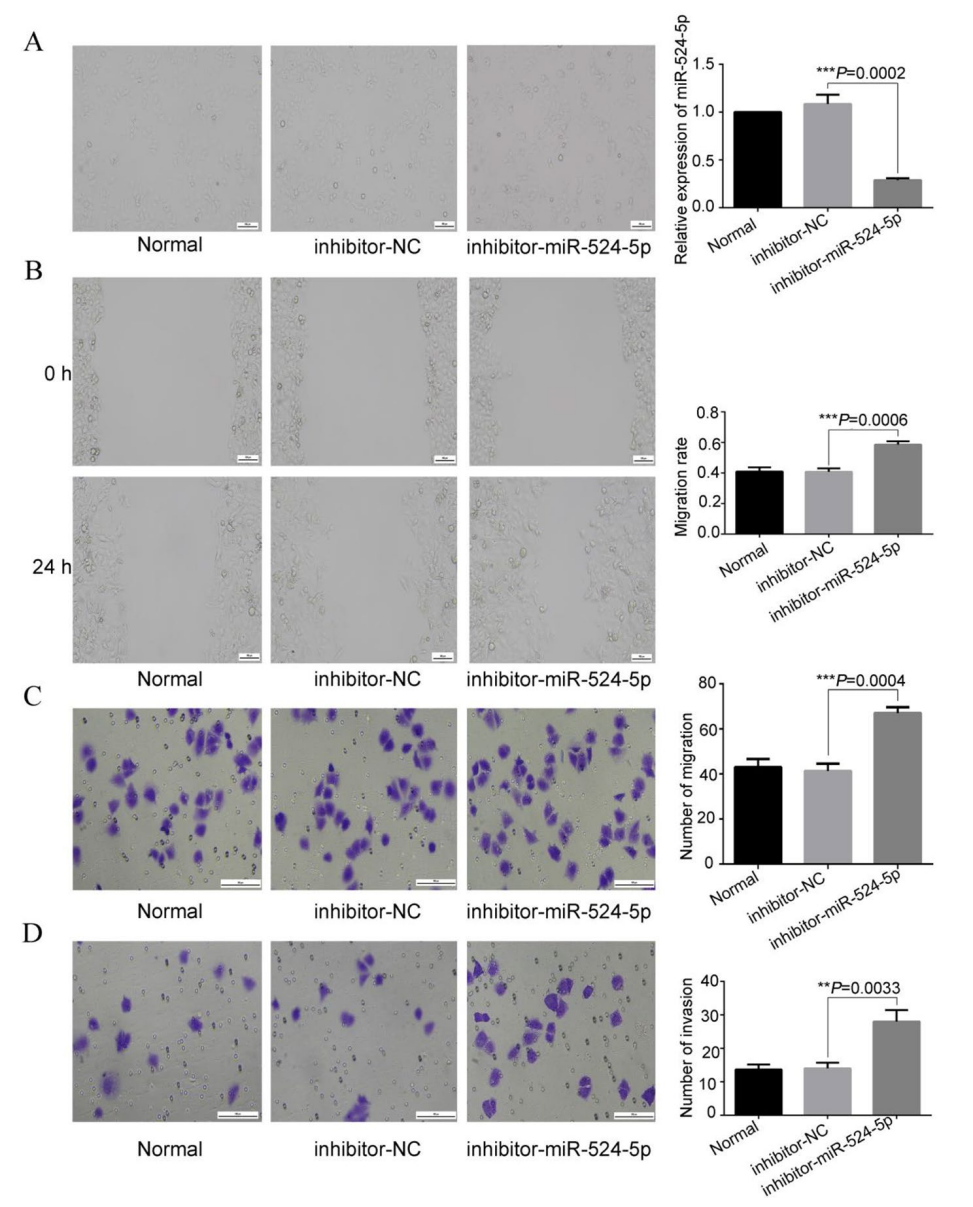
Effect of miR-524-5p on the wound healing, migration, and invasion of ISK cells. (**A**) Transfection with miR-524-5p inhibitors resulted in the lower expression of miR-524-5p in ISK cells. miR-524-5p inhibitors increased the (**B**) wound healing capacity, (**C**) migration ability, and (**D**) invasion ability of ISK cells. *P* < 0.05 indicates a statistically significant difference. Data are expressed as the mean ± SD of three independent experiments. Magnification of microscope: 200 ×. The number of cells: 4 × 10^5^/well. SD, standard deviation

First, miR-524-5p mimic and miR-524-5p inhibitor were transfected into ISK cells. Cell viability (*P* = 0.009; Fig. 6C) decreased significantly in miR-524-5p mimic-transfected ISK cells compared with NC-transfected cells. Meanwhile, Cell viability (*P* = 0.0045; Fig. 6C) increased significantly in miR-524-5p inhibitor-transfected ISK cells compared with NC-transfected cells. The transcription levels of miR-524-5p were confirmed via qRT-PCR. Compared with the NC-transfected cells, miR-524-5p mimic–transfected cells showed significantly increased *miR-524-5p* expression (*P =* 0.0005; Fig. 7A). Wound healing (*P* = 0.0013; Fig. 7B), migration (*P* < 0.0001; Fig. 7C), and invasiveness (*P* = 0.0009; Fig. 7D) decreased significantly in miR-524-5p mimic-transfected ISK cells compared with NC-transfected cells. In addition, miR-524-5p inhibitor-transfected cells showed significantly decreased miR-524-5p expression (*P* = 0.0002; Fig. 8A). Wound healing (*P* = 0.0006; Fig. 8B), migration (*P* = 0.0004; Fig. 8C), and invasiveness (*P* = 0.0033; Fig. 8D) increased significantly in miR-524-5p inhibitor-transfected ISK cells compared with NC-transfected cells.

### Mir-524-5p regulates the expression of four EC-related genes

**Fig. 9:**
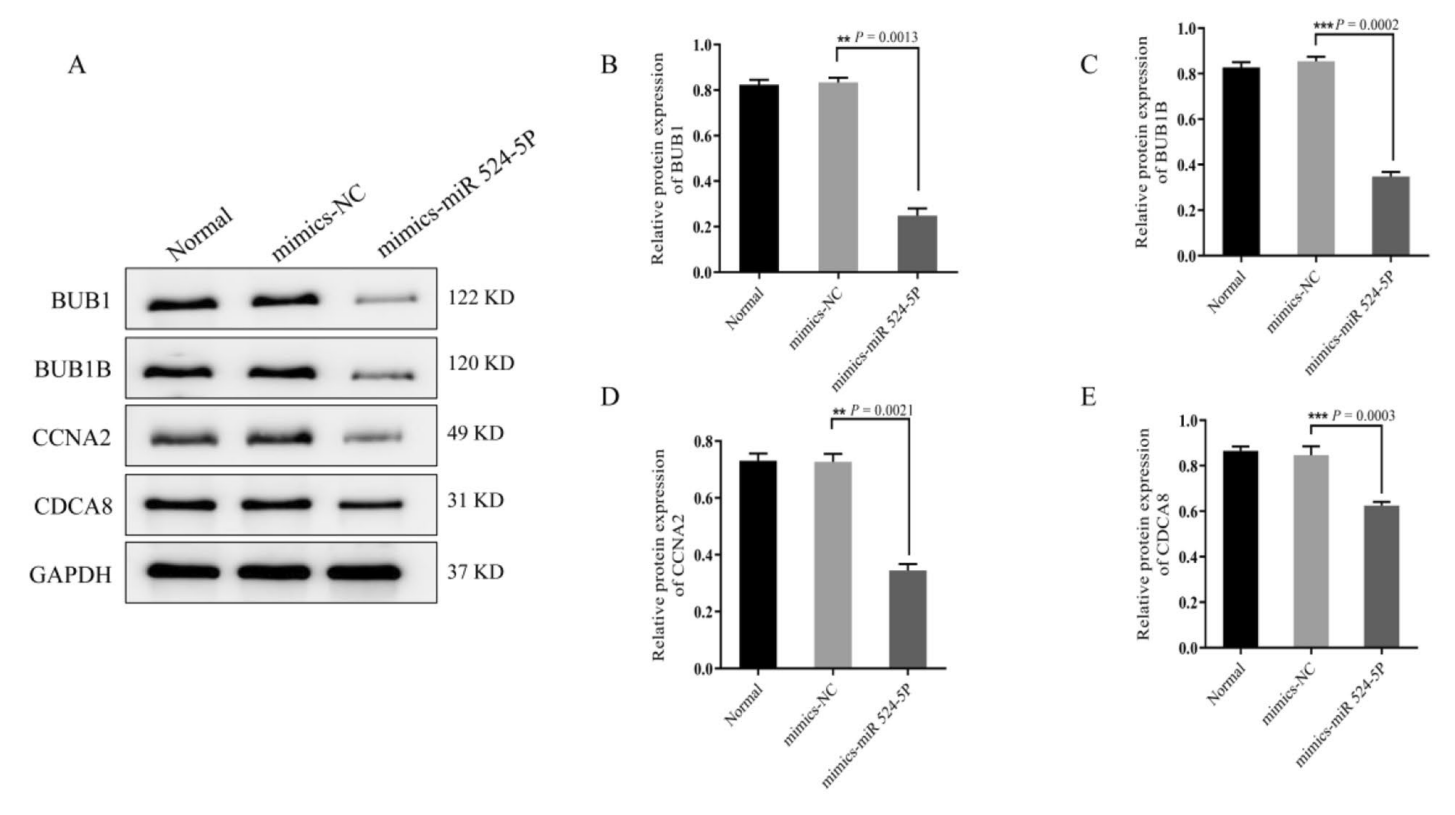
Effect of miR-524-5p on BUB1, BUB1B, CCNA2, and CDCA8 protein expression. (**A**–**E**) miR-524-5p reduced BUB1, BUB1B, CCNA2, and CDCA8 expression. Compared with the control group, the overexpression group showed reduced BUB1, BUB1B, CCNA2, and CDCA8 expression. *P* < 0.05 was considered statistically significant. Data are expressed as the mean ± SD of three independent experiments. The number of cells: 4 × 105/well. SD, standard deviation

**Fig. 10:**
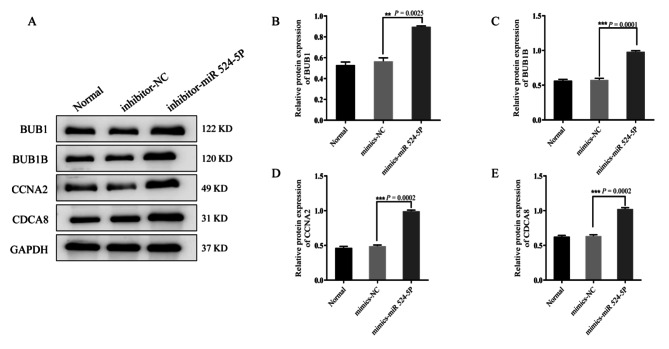
Effect of miR-524-5p on BUB1, BUB1B, CCNA2, and CDCA8 protrein expression. (**A**–**E**) miR-524-5p inhibitors increased BUB1, BUB1B, CCNA2, and CDCA8 expression. Compared with the control group, the inhibitor miR-524-5p showed increased BUB1, BUB1B, CCNA2, and CDCA8 expression. *P* < 0.05 was considered statistically significant. Data are expressed as the mean ± SD of three independent experiments. The number of cells: 4 × 10^5^/well. SD, standard deviation

Western blot analysis showed that the levels of BUB1 (*P* = 0.0013), BUB1B (*P* = 0.0002), CCNA2 (*P* = 0.0021), and CDCA8 (*P* = 0.0003) in miR-524-5p mimic-transfected ISK cells were significantly lower than that in the mimics-NC group (Fig. 9A–E). The protein expression levels of BUB1 (*P* = 0.0025), BUB1B (*P* = 0.0001), CCNA2 (*P* = 0.0002) and CDCA8 (*P* = 0.0002) in miR-524-5p inhibitor were significantly higher than those in the inhibitor-NC group by using western blot (Fig. 10A–E).

## Discussion

EC biomarkers are valuable for screening high-risk patients, stratifying risk among patients, developing individualized treatment options, and assessing prognoses. Currently, the etiology of EC remains unclear. Type I estrogen-dependent EC is associated with *PTEN* mutation and inactivation, while type II non-estrogen-dependent EC may be related to *P53* mutation, *P16* (cyclin-dependent kinase inhibitor 2 A) inactivation, E-cadherin inactivation, and *HER2* (human epidermal growth factor receptor 2) overexpression. Microarray technology is effective in detecting new biomarkers of the disease, providing clues on alternative strategies to assess and manage EC. EC and the tumor microenvironment can modulate immune responses. In gynecologic cancers, EC showed the highest overexpression of programmed cell death 1 (PD-1, CD279) and programmed cell death ligand 1 (PD-L1, CD274): 40–80% in endometrioid carcinoma, Serous carcinomas accounted for 10–68% of tumors, and clear cell tumors accounted for 23–69%, respectively [36]. PD-1 is a cell surface protein encoded by the PDCD1 gene, especially expressed on the surface of activated B and T lymphocytes. Cytotoxic T lymphocyte antigen 4 (CTLA-4), lymphocyte activation gene 3 (LAG-3), and IDO may also be upregulated in POLE tumors [37]. Regulatory T lymphocyte infiltration was an independent prognostic factor in mutated TP53 tumors. Some studies have shown that although POLE and MSI-H tumors have the strongest immune response, some serous and low copy number tumors also have a strong immune response [38]. These suggest that patients opting for immunomodulatory therapy may not be limited to MMRd to achieve the highest sensitivity and specificity.

The current study used EC-related transcriptome data from 587 samples. DEGs and their functional annotations, as well as hub genes, were screened. Analysis of the biological functions and signaling pathways showed that the identified hub genes were mainly related to cell division, cell adhesion, angiogenesis, cell cycle progression, cell growth, calcium signaling, cGMP-PKG signaling pathway, cAMP signaling pathway, Rap1 signaling pathway, Ras signaling pathway, and P53, which are closely related to EC progression and development. It was reported that 4 miRNAs can be used to classify high- and low-risk patients with EC [39]. In particular, the expression of 9 long noncoding RNAs was reported to be significant for the survival rate in patients with EC [40]. *P53* mutation was suggested to lead to serious carcinoma diagnosis [41]. Overall, EC-related signaling pathways have been shown to be associated with the p53 signaling pathway, cell cycle, PI3K-Akt, and MAPK, among others.

The present study investigated 4 survival-related hub genes (*BUB1, BUB1B, CCNA2*, and *CDCA8*), as well as their encoded proteins, and determined that they all play important roles in EC pathogenesis. *BUB1* encodes a serine/threonine protein kinase that phosphorylates mitotic checkpoint complexes, activates spindle checkpoints, promotes the formation of TGFBRI/II receptor complexes, and mediates TGFβ-dependent epithelial-mesenchymal transition, thereby promoting cell migration and invasion. Analyses of TCGA data and immunohistochemical results of pathological tissue samples indicated that *BUB1* is highly expressed in EC. Taken together, these findings elucidate the role of *BUB1* in promoting tumorigenesis and development. BUB1B, CCNA2, and CDCA8 are rarely reported in association with EC. BUB1B, also known as BUBR1, is a functional protein at the checkpoint [42], which contributes to tumor development and progression [43]. CCNA2 was found to be expressed in adenocarcinoma of lung and oral cancer cells [44]. Our study also indicated that its level in EC was higher than that in normal uterine tissue. CCNA2 has an important role in promoting G1-S cell cycle progression [45] by facilitating CCND1 regeneration and participating in processes such as chromosome instability, cancer cell function, and cell maturation [46]. CDCA8 overexpression was reported in human breast, gastric, and lung cancers, implying that CDCA8 is essential for the growth and development of certain cancers. A subset of mutations associated with *BUB1* and *CCNB1* [47] was also correlated with CDCA8. Our research indicated that the presence of a mutual regulatory relationship between *BUB1*, *BUB1B*, *CCNA2*, and *CDCA8*, which may provide a theoretical basis for clarifying EC pathogenesis and developing appropriate therapies.

The miRNA database (TargetScan) was used to determine the molecular mechanism that regulates the occurrence and development of EC, which revealed that *BUB1*, *BUB1B*, *CCNA2*, and *CDCA8* were coregulated by miR-524-5p. This recently discovered miRNA has been described to be abnormally expressed in tumors. However, its specific role in EC remains unclear. The current study determined that miR-524-5p regulates the EC hub genes *BUB1, BUB1B, CCNA2*, and *CDCA8*. Moreover, miR-524-5p could inhibit wound healing, migration, and invasion of EC cells by regulating the levels of *BUB1*, *BUB1B*, *CCNA2*, and *CDCA8* in patients with EC.

Our research has some limitations. First, our analysis of gene survival is based on a public database. Additionally, our research included a relatively small sample size, and patients with EC should be followed up to further analyze the clinical value of *miR-524-5*p, *BUB1*, *BUB1B*, *CCNA2*, and *CDCA8*.

## Conclusions

Our study findings indicate that *BUB1*, *BUB1B*, *CCNA2*, and *CDCA8* are highly expressed in EC. Furthermore, miR-524-5p reduces the migration and invasion of EC cells and the expression of *BUB1*, *BUB1B*, *CCNA2*, and *CDCA8*. Therefore, these results suggest that miR-524-5p can regulate *BUB1*, *BUB1B*, *CCNA2*, and *CDCA8*, thereby contributing to the development of EC. Thus, miR-524-5p, as well as *BUB1*, *BUB1B*, *CCNA2*, and *CDCA8*, may be clinically relevant biomarkers for the diagnosis and treatment of EC.

### Electronic supplementary material

Below is the link to the electronic supplementary material.

Supplementary Material 1

Supplementary Material 2

## Data Availability

The datasets used and/or analyzed during the current study are available from the corresponding author on reasonable request. In this study, Because we don’t use KEGG IMAGE in our diagram. Therefore, Kanehisa Laboratories allows us to use the KEGG database directly. We don’t have to get KEGG’s copyright license from them.
