# Supplementary material for: BUB1, BUB1B, CCNA2, and CDCA8, along with miR-524-5p, as clinically relevant biomarkers for the diagnosis and treatment of endometrial carcinoma
Source: BMC Cancer. 2023 Oct 18;23:995. doi: 10.1186/s12885-023-11515-9 (PMC10585751; doi:10.1186/s12885-023-11515-9)
Supplement: Supplementary file 1 — Supplementary Material 1 [file 12885_2023_11515_MOESM1_ESM.pdf]

BUB1

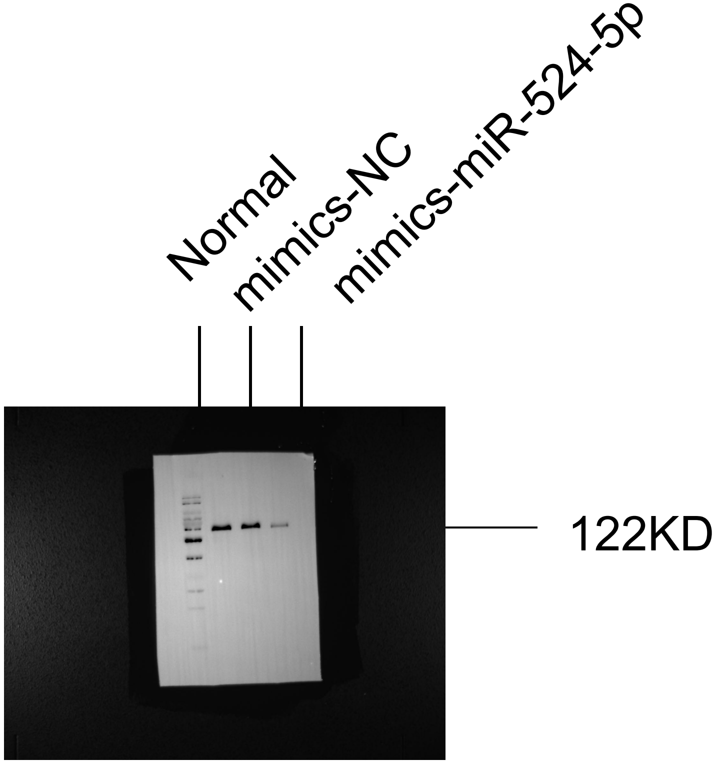

The original Western Blot images of BUB1 in Figure 9. From left to right: Normal, mimics-NC, mimics-miR-524-5p. The expected molecular weight is 122 kDa.

BUB1B

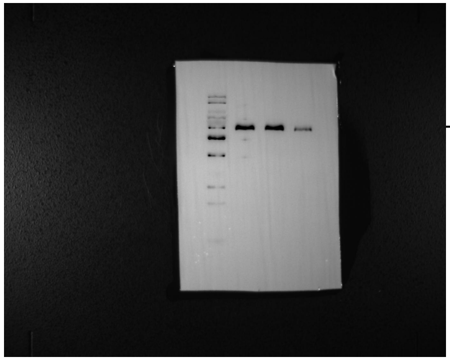

The original Western Blot images of BUB1B in Figure 9. From left to right: Normal, mimics-NC, mimics-miR-524-5p. The expected molecular weight is 120 kDa.

CCNA2

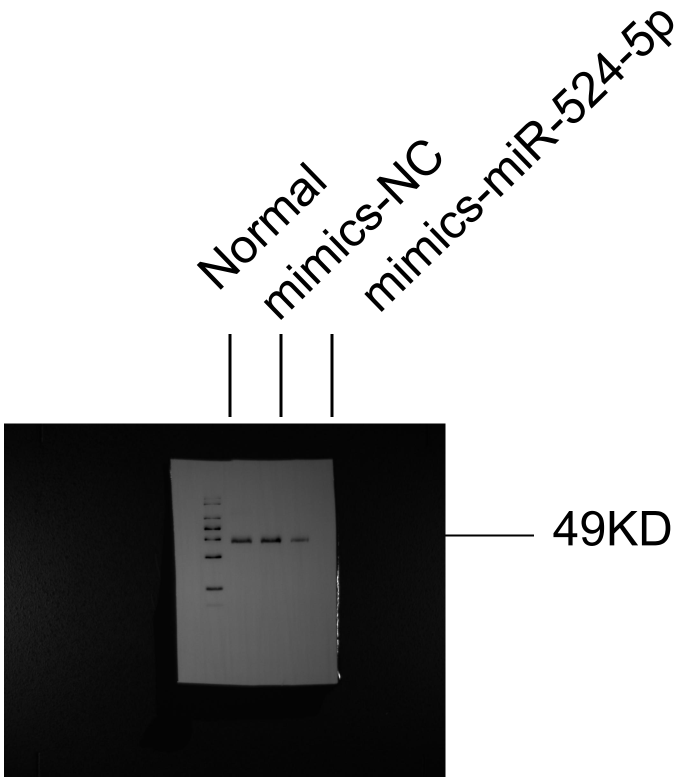

The original Western Blot images of CCNA2 in Figure 9. From left to right: Normal, mimics-NC, mimics-miR-524-5p. The expected molecular weight is 49 kDa.

CDCA8

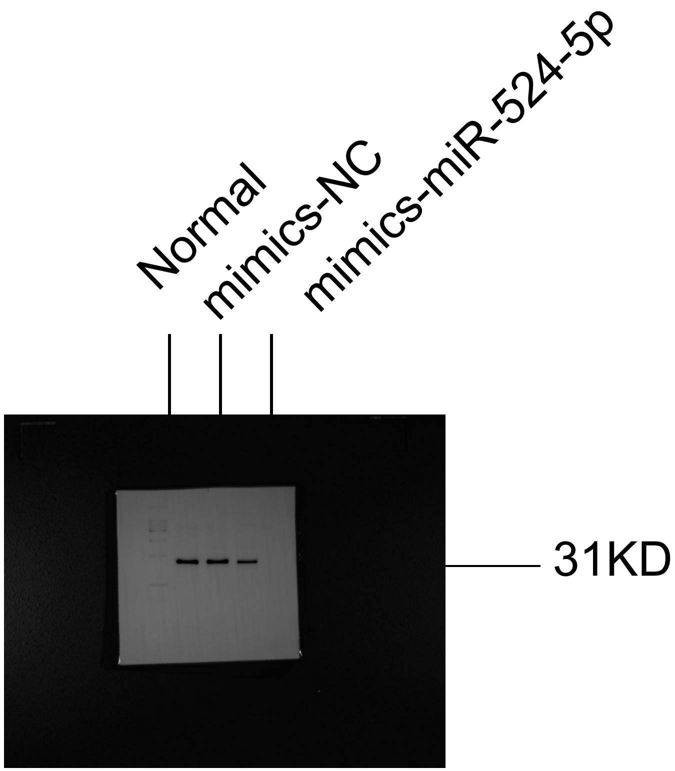

The original Western Blot images of CDCA8 in Figure 9. From left to right: Normal, mimics-NC, mimics-miR-524-5p. The expected molecular weight is 31 kDa.

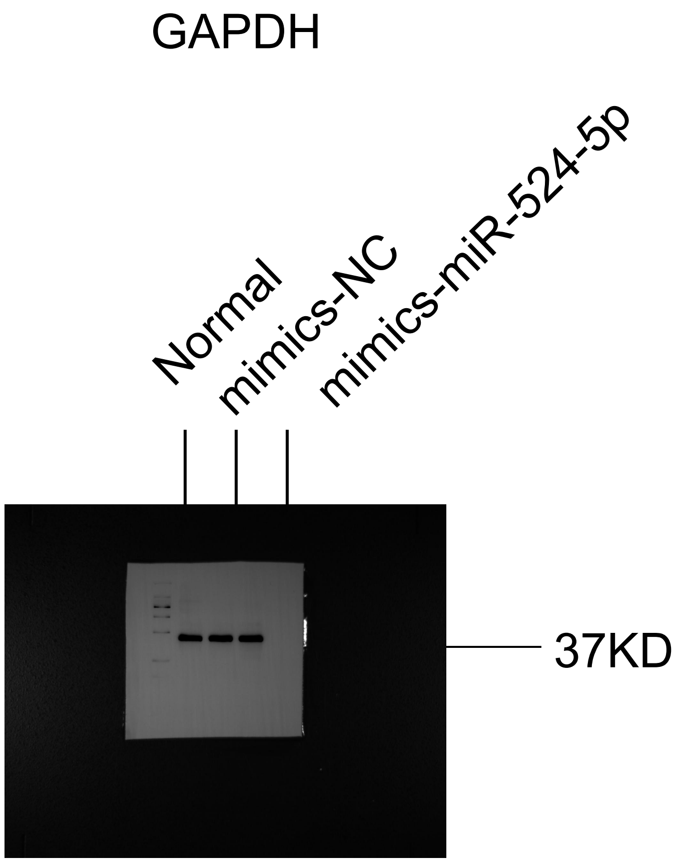

The original Western Blot images of GAPDH in Figure 9. From left to right: Normal, mimics-NC, mimics-miR-524-5p. The expected molecular weight is 37 kDa.

BUB1

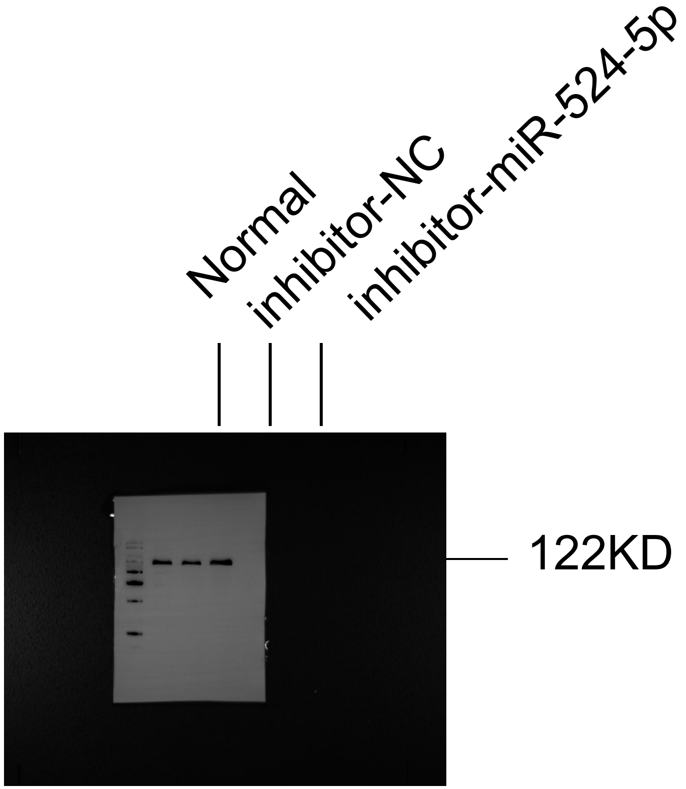

The original Western Blot images of BUB1 in Figure 10. From left to right: Normal, inhibitor-NC, inhibitor-miR-524-5p. The expected molecular weight is 122 kDa.

BUB1B

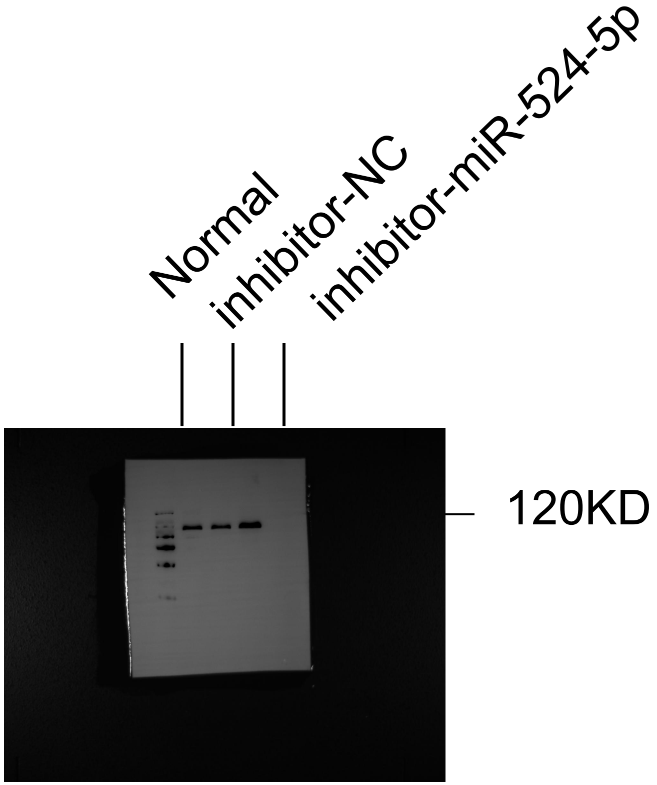

The original Western Blot images of BUB1B in Figure 10. From left to right: Normal, inhibitor-NC, inhibitor-miR-524-5p. The expected molecular weight is 120 kDa.

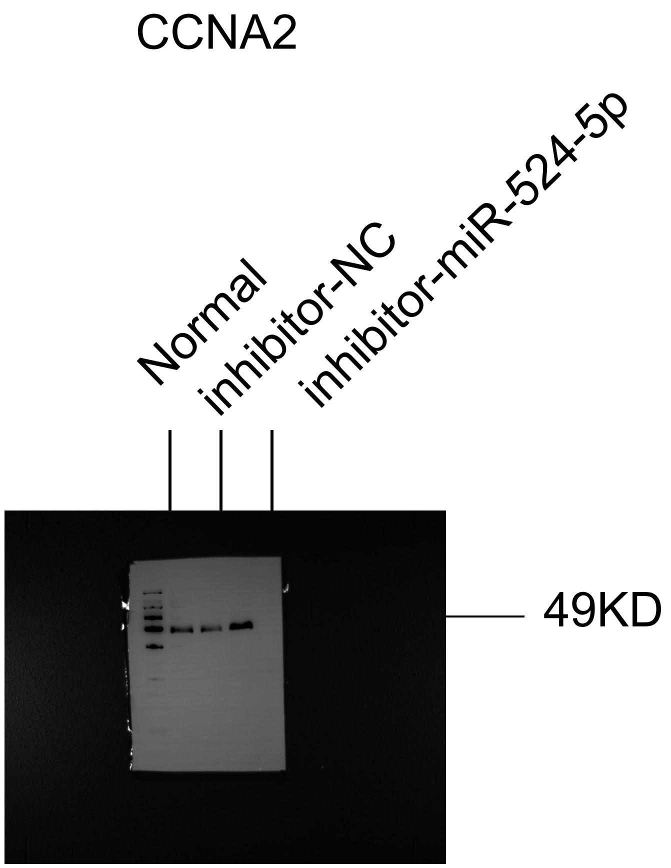

The original Western Blot images of CCNA2 in Figure 10. From left to right: Normal, inhibitor-NC, inhibitor-miR-524-5p. The expected molecular weight is 49 kDa.

CDCA8

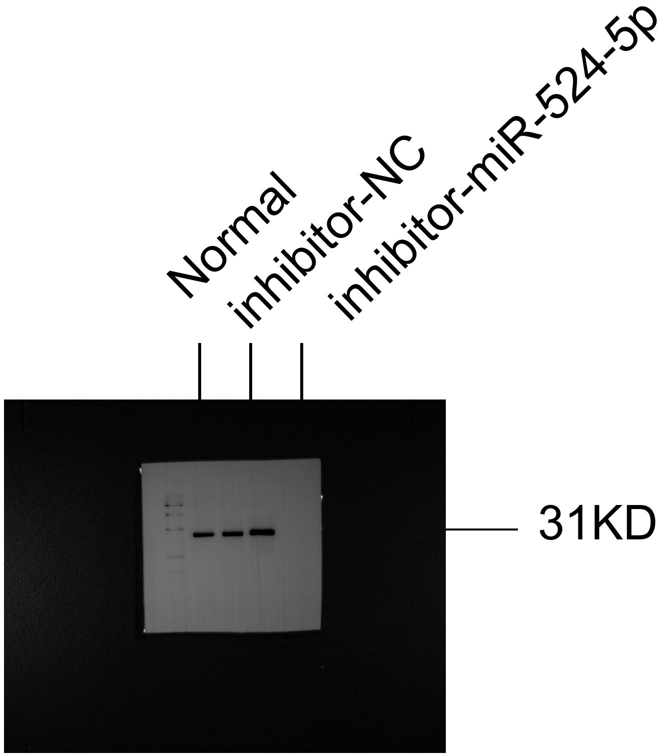

The original Western Blot images of CDCA8 in Figure 10. From left to right: Normal, inhibitor-NC, inhibitor-miR-524-5p. The expected molecular weight is 31 kDa.

GAPDH

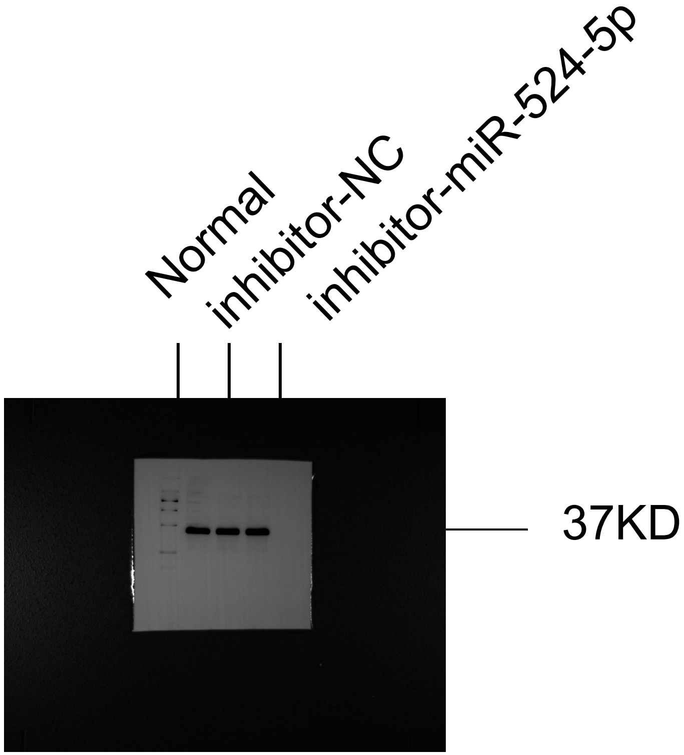

The original Western Blot images of GAPDH in Figure 10. From left to right: Normal, inhibitor-NC, inhibitor-miR-524-5p. The expected molecular weight is 37 kDa.
