# Supplementary material for: BUB1, BUB1B, CCNA2, and CDCA8, along with miR-524-5p, as clinically relevant biomarkers for the diagnosis and treatment of endometrial carcinoma
Source: BMC Cancer. 2023 Oct 18;23:995. doi: 10.1186/s12885-023-11515-9 (PMC10585751; doi:10.1186/s12885-023-11515-9)

Supplementary Figure 1 ENCORI database (http://starbase.sysu.edu.cn/index.php) is used to predict the target sites between miR-524-5p and BUB1, miR-524-5p and BUB1B, miR-524-5p and CCNA2, miR-524-5p and CDCA8.


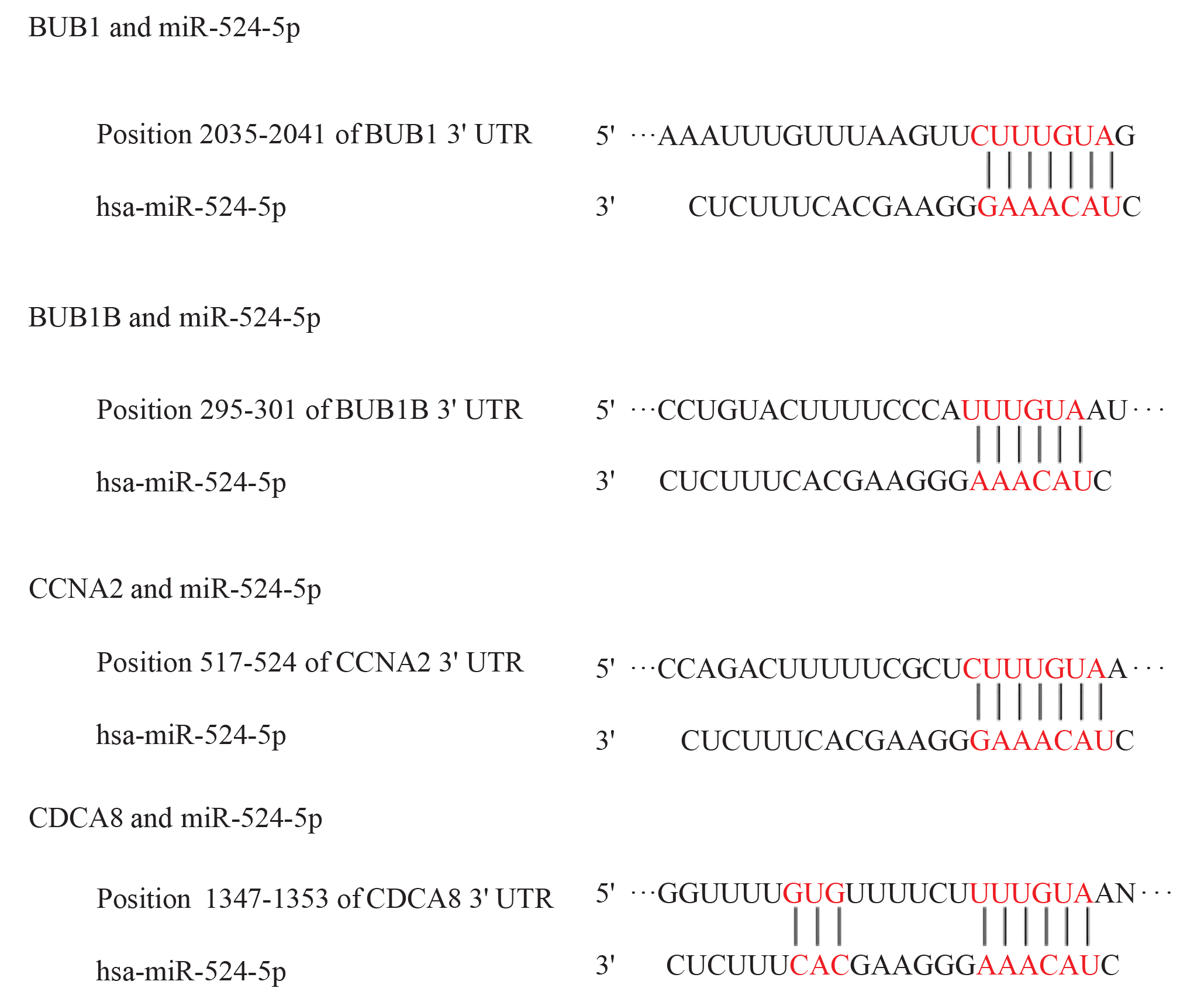

Supplement: Supplementary file 2 — Supplementary Material 2 [file 12885_2023_11515_MOESM2_ESM.doc]
